# Notes and News

**Published:** 1889-01-05

**Authors:** 


### Notes and News.

Collected and Communicated.

We are obliged to delay the continuance of Voyages in Vacation until next week, when a graphic description of the Kremlin, Moscow, will be given.

An outbreak of small-pox has occurred at Dundee. The disease was brought into the city by a sailor from Oran, Algeria. A sanitary officer who removed the sailor to the hospital was attacked, as was also the mate of the ship. The latter died. One of the crew of another vessel from Oran was removed to the hospital.

Dr. Symons, of Kingskerswell, near Torquay, died on December 23rd at his residence, from injuries received on the previous day. When returning home in a dogcart, the horse became frightened at the opening of an umbrella and bolted down a steep hill near Newton Abbot. The shaft broke at the bottom of the hill, and Dr. Symons was thrown out.

Tiverton Infirmary seems to have an energetic secretary. Under the management of Mr. Fisher, a concert was given on December 20th, which brought in £54, which is not at all bad for such a small town. The infirmary was suffering from monetary embarrassments, which this sum will relieve. Amongst the performers were Miss Chichester, Herr Otto Milani, Lady Margaret Wallop, Lady Heathcoat-Amory, and others.

The managers of All Saints' Convalescent Home, Eastbourne, has gone the length of refusing admittance to *Truth*. Now, though no lovers of society papers, still we are all bound to admit that *Truth* is a good friend to our hospitals, not only by the toys it collects and distributes every year, but because it does not hesitate to expose charity swindles which are far more numerous than many suppose. We should, therefore, like to hear if the decision is final, and on what grounds it has been arrived at?

A series of public conferences is being held at Manchester under the auspices of the Manchester and Salford Sanitary Association, to consider the high death-rate and the evil influence of black smoke. At the last meeting, Dr. Morgan said Manchester was the most unhealthy town in England, and it was owing to its filthy atmosphere, which was made filthy by manufacturers who lived from five to thirty miles distant. The atmosphere contaminated the blood, produced bronchial and throat diseases, lessened the capacity for work, and drove men to drink. He moved: "That, in view of the continued high death-rate, this meeting calls upon the City Council to carry out the suggestions made at the conference in the Memorial Hall last June, and further urges that special attention be given to the influence of polluted atmosphere on the health of the city, with a view to a more stringent enforcement of powers held by them of dealing with the smoke nuisance." Dr. Scott seconded the motion, which was carried. Sir Henry Roscoe, M.P., Dr. Arthur Ransome, Dr. John Tresh, and others, wrote very strongly on the subject.

Dr. Allinson is doing his best to cry down the wisdom of generations and to cry up the one-sided doctrines of vegetarianism. Because Dr. Allinson can live on bread and water and feel few bad effects is no reason why we should despise the good things the gods provide. That meat contains but little nourishment; that 80 or 90 per cent. of meat brought to the London market is diseased; that meat-eating causes persons to have tape-worms 30 ft. long are indeed startling facts. But even facts will lend themselves to delusions when cleverly handled.

Of all the many hospitals where pleasure was to be found at Christmas, we would have chosen the General Hospital, Nottingham, had we been a patient. There was the usual dramatic entertainment in which three of the resident staff took part, there were the usual decorations and extras for dinner, but the specialité was that all the male patients had two or three good cigars given them and were allowed to smoke during the afternoon and evening. Not pipes, mind you, but cigars! No wonder all the patients enjoyed the day very much, and exclaimed next morning that they had 'slept the night right through for a wonder!'

In our "Philanthropist's Vade Mecum" of last week we omitted one charity of great worth and of great need: the Aftercare Association, for poor and friendless female convalescents on leaving asylums for the insane. During the past year 48 cases have been before the Committee, of which 41 have been accepted, 6 declined as not suitable, and 1 withdrawn. The convalescents are sent to cottage homes in the country, and when necessary, provided with suitable clothing. Very often situations are provided for those who can work, and they are fairly started in the world once more. The Princess Christian is patroness of the association, and Dr. Hack Tuke, Miss Davenport Hill, and other well-known names are on the council. The secretary is Mr. H. Thornhill Roxby, Arden Lea, The Drive, Walthamstow.

On December 10th Dr. B. W. Richardson read a paper before the Medical Society of London on the absolute signs of death. Nothing could be more appropriate, considering the number of "resurrection" stories now going the round of the press. The most extraordinary of these stories comes from Bolton, where a young man took an overdose of morphia, and was pronounced dead by two doctors. The body was laid out, but the following morning signs of animation were observed, and for a few hours the youth recovered semi-consciousness, only to die in earnest in the end. Were all Dr. Richardson's ten proofs of death, including absence of red blotch on the skin after injection of ammonia, absence of rust on a needle plunged into the tissues, &c., tried in this doubtful case? There is nothing frightens the public more than these stories, and the fewer we have of them the better.

It is rumoured that rather than give back the big fee he has received for attending the Nawab of Rampur, Dr. Freyer will resign his Government commission. The *St. James's Gazette* gives the following particulars about big fees: "When Dr. Beaumont attended the Nizam, it was proposed to give half a lakh; but, on the Government of India interposing, the fee was limited to 10,000 rupees. Dr. Caley received 10,000 rupees for an operation on the Maharajah of Benares, having only remained one day with the patient; Dr. Macnamara got half a lakh of rupees for operating on the Maharajah of Jeypore; and the grandfather of the present Nawab of Rampur paid a native physician one lakh for attendance in an illness from which he never completely recovered. In none of these cases did Government interfere, and there would seem to be even less cause for interference in Dr. Freyer's case, where the illness was more complicated, the attendance more protracted, and the recovery complete."

THE following vacancies are announced this week, the amount of salary in each case being given after the name of the institution :—

**Resident Medical Officers.**

Worcester County and City Lunatic Asylum. Third Assistant Medical Officer.—£100, board, etc.
County of Clare Infirmary. Surgeon.—£94, and residence.
Durham County Asylum. Second Assistant Medical Officer.—£150, board, etc.
City of London Hospital for Diseases of the Chest. Clinical Assistant.
Derbyshire General Infirmary. Assistant House Surgeon.—Board, etc.
Darenth Schools for Imbecile Children. Assistant Medical Officer.—£120, board, etc.
Hospital for Sick Children, Great Ormond Street. House Surgeon.—£50, board, etc.
Radcliffe Infirmary, Oxford. House Surgeon.—£80, board, etc.
Nottingham General Dispensary. Senior Surgeon.—£180, coal, etc.

**Non-resident Medical Officers.**

Guy's Hospital Six Assistant Dental Surgeons; Lecturers on Dental Anatomy and Physiology, and Dental Mechanics; an Anæsthetist; and a Tutor for the Dental Students.
Pendleton Branch Dispensary. Honorary Medical Officer.

---

THE Surgical Aid Society was established in 1862, and was for many years the only society through which all surgical appliances and artificial limbs could be obtained. Last year it gave 11,819 surgical appliances to the afflicted poor. The patients are assisted on the recommendation of subscribers, the committee making special grants towards expensive instruments. The secretary has power to give immediate help to urgent cases without requiring subscribers' letters. Water beds and invalid carriages are lent to the poor. The useful work of the society is only limited by want of funds, which are greatly needed, and may be sent to the secretary, Mr. Wm. Tresidder, at the office, Salisbury Square, E.C.

---

AT the Consumption Hospital, Brompton, Christmas was observed in right festive fashion. The galleries and wards presented a very bright and picturesque appearance, appropriate decorations being put up in endless variety by the nurses and patients, the evergreens having been kindly sent by the Marquis of Abergavenny, the Countess of Darnley, Countess Sydney, Lady Northbourne, and other friends. The pretty chapel was also tastefully decorated by Mrs. Hughes-Owen. Very early on Christmas morning a party of the choir and nurses visited the various wards, and sang a selection of carols with charming effect. After morning service in the chapel, conducted by the Rev. J. Hughes-Owen, the chaplain, the patients sat down to dinner, and much enjoyed the Christmas fare of turkeys and plum pudding, the former liberally provided by Mr. Bevan, Mr. Robert Gillespie, Sir T. Sebastian Bazley, the Rev. W. T. Gordon, the Rev. W. B. Morris, and Mr. D. B. Chapman. A chest of oranges from Mr. Bevan, fruit from other friends, and a cask of wine from Messrs. Basil Woodd and Sons furnished the dessert. The evening was enlivened by music, singing, and games, the excellent and kind-hearted Lady Superintendent and Resident Medical Officers (Miss Florence Abbott, Dr. H. D. Waugh, and Mr. Herbert Taylor), with the House Physicians and Sisters, exerting themselves most energetically to promote the enjoyment of the inmates, in which labour of love they were assisted by several ladies and gentlemen. The general festivities culminated on Friday evening in a huge Christmas tree, provided by the kindness of the Miss Heddys, who, with Miss Abbott, bestowed much time and labour in collecting, arranging, and distributing the numerous gifts sent by the physicians and many friends, including the editor of *Truth*. The tree was gaily decked with flags and Chinese lanterns. The spacious entertainment hall, a special feature of this hospital, was filled with the patients, nurses, and servants, and many visitors. A feature of the evening was the appearance of Dr. Russell (a former house physician) as "Father Christmas," who emerged from a mimic snow cavern, glittering with icicles, much to the delight of the audience. After the distribution, there were music and songs by Dr. Anderson Smith and Dr. Russell, and the proceedings appropriately concluded with a vote of thanks to the friends who had contributed in so many ways to the enjoyment of all.

A CHRISTMAS TREE FETE was given on the 27th ult. to the patients of the National Hospital for Paralysed and Epileptic, Queen Square. The out-patient room was utilised, and over a hundred patients were assembled there, round a large Christmas tree that reached from floor to ceiling. The distribution of gifts took place on the lottery principle, and every one—nurses, doctors, visitors, and all—participated in it. There were shouts of delight from grown-up as well as childish throats when the tree was being despoiled, not altogether caused by the fitness of the gifts to the situation of those who received them; at least, this would scarcely account for the delight that was manifested when the secretary received a Noah's ark, and the matron was awarded a doll. Good care was taken, however, that the patients should receive presents they could make use of, and in their happy, smiling faces it was difficult to trace any signs of the serious diseases from which many of them were suffering. The credit of this most successful entertainment is due to the lady superintendent, Miss East, and the sisters.

---

THE Christmas trees at the Evelina Hospital for Children were lighted at three o'clock on Friday, December 28th, and from that time till six there was rejoicing and excitement among the small patients there, and it is just possible that the nurses found it difficult to soothe some of their patients to sleep afterwards, the latter having so much to do in comfortably settling for the the night the dolls, horses, cavalry regiments, and other treasures of which they had recently become possessed.

---

UNIVERSITY COLLEGE HOSPITAL was unfortunate in having its fête on the foggy 31st, which prevented visitors from attending; but the weather within was brighter than that without. The patients saw the old year out with lighter hearts than they had expected, and the hospital looked so bright that no one would have believed under how heavy a load of debt it was carrying on its beneficent work.

---

THE Seamen's Hospital (late Dreadnought) made holiday for a week. On Christmas Day there was a seasonable dinner of turkey and plum-pudding, with a dessert of pipes and tobacco, which were for once allowed to be used in the wards. A conversazione for the nurses took place on the 2nd inst., and a dramatic performance for the amusement of the patients on the 3rd, while the festivities were brought to a close on the 4th by a Christmas tree for all the children who have been treated by the society during the year. A pretty comprehensive series of entertainments, which has given a generous amount of pleasure.

---

THE first thing that met the eye on entering the Queen Victoria ward at the London Hospital on New Year's Day was a regiment of small red backs. The heads pertaining to these were turned with one accord to the Christmas Tree at the end of the ward, where candles were being lighted. Intense silence prevailed while this was going on, but when the distribution of gifts began there was a moderate—a very moderate—amount of impatience. The toys were handsome and numerous, a considerable number of dolls having been sent by H.R.H. Princess Christian, and before very long everyone had a selection that seemed eminently suited to their individual preferences, which had evidently been consulted. At four o'clock the cots were moved from the post of vantage whence they had seen the tree back to their usual stations by the wall, and the numerous visitors, who had seemed almost as pleased as the children at the proceedings, gathered in the nursing home to drink tea and talk over the proceedings with much satisfaction.

